# Topical Neoagarohexaose from Red Algae Alleviates Acute MSU-Induced Paw Inflammation Accompanied by Reduced *Ccl2*-Associated Macrophage Chemotaxis

**DOI:** 10.3390/cimb48101074

**Published:** 2026-10-17

**Authors:** Chaocheng Wu, Yating Du, Shuting Wang, Xinyue Chang, Huangting Li, Runying Zeng, Nan Wu

**Affiliations:** 1 Technology Innovation Center for Exploitation of Marine Biological Resources, Third Institute of Oceanography, Ministry of Natural Resources, Xiamen 361005, China; 2 Fujian Provincial Key Laboratory of Island Conservation and Development, Island Research Center, Ministry of Natural Resources, Pingtan 350400, China

**Keywords:** neoagarohexaose, red algae, MSU-induced paw inflammation, gout, topical administration, CCL2

## Abstract

Red algae-derived bioactive oligosaccharides have drawn increasing attention in drug discovery, yet their therapeutic potential in crystal-induced inflammation remains underexplored. Here, we investigated the anti-inflammatory effects and underlying mechanisms of neoagarohexaose (NA6), a well-defined agarose-derived oligosaccharide, in a mouse model of acute monosodium urate (MSU)-induced paw inflammation—a widely used surrogate of acute gout flares—and in RAW264.7 macrophages. Topical application of NA6 significantly alleviated paw swelling and subcutaneous tissue damage and was accompanied by reduced circulating myeloperoxidase activity and pro-inflammatory cytokines (TNF-α, IL-1β, and IL-6) at 24 h; under the present conditions, the high-dose NA6 group did not differ significantly from the diclofenac emulgel group. Immunofluorescence and qRT-PCR analyses confirmed reduced cytokine expression in paw tissues. Transcriptomic profiling and network analysis identified *Ccl2* as the chemokine most strongly suppressed following NA6 treatment. In vitro, NA6 reduced MSU-induced *Ccl2* expression and CCL2 secretion in macrophages, which was accompanied by reduced chemotactic migration, an effect partially reversed by recombinant CCL2 supplementation. Collectively, these findings show that topical NA6 alleviates acute MSU-induced paw inflammation and that this effect is accompanied by reduced CCL2 production and CCL2-associated chemotactic activity, supporting NA6 as a marine-derived candidate worthy of further investigation for the topical management of gout flares.

## 1. Introduction

Gouty arthritis (GA) is an inflammatory joint disease caused by monosodium urate (MSU) crystal deposition in joints and surrounding tissues [1], primarily manifesting as recurrent episodes of joint redness, swelling, warmth, and pain [2,3]. Without timely intervention, it may progress to chronic joint damage or even secondary osteoarthritis, severely impacting patients’ quality of life [4]. Moreover, GA is often associated with metabolic syndrome, cardiovascular disease, and renal dysfunction, extending its impact beyond local joint inflammation. Current pharmacological treatments, including nonsteroidal anti-inflammatory drugs (NSAIDs), colchicine, and glucocorticoids, provide effective symptomatic relief but are limited by gastrointestinal, renal, and cardiovascular adverse effects, and a considerable proportion of patients experience inadequate responses or contraindications to these agents [5,6,7]. Therefore, the development of additional anti-inflammatory agents with favorable safety profiles remains an important research priority.

The pathogenesis of GA is driven by a self-perpetuating cycle of inflammation and chemokine-mediated cellular recruitment. MSU crystal deposition acts as a potent danger signal, triggering robust activation of resident immune cells and inducing the release of pro-inflammatory mediators [8,9,10]. Among the key mediators of this process, chemokines play an essential role in recruiting inflammatory cells to the site of crystal deposition. In particular, CCL2 is critically involved in the pathogenesis of GA by promoting the infiltration of monocytes and macrophages into inflamed tissues [11,12]. Upon MSU stimulation, resident macrophages and synovial cells secrete large amounts of CCL2, which promotes the recruitment of circulating monocytes and amplifies the local inflammatory response [13]; MSU-recruited monocytes have also been shown to differentiate into CCL2-producing pro-inflammatory macrophages in murine models of gout [14]. Thus, modulating CCL2-mediated chemotaxis represents a promising strategy for interrupting this pathogenic cascade.

Neoagarohexaose (NA6) is a functional oligosaccharide derived from agarose polysaccharides sourced from red algae through agarase hydrolysis [15]. Recent studies have reported that NA6 exhibits antiviral [16] and immune-enhancing effects [17]—the latter involving context-dependent TLR4 agonism in dendritic cells—and inhibits β-amyloid-induced oxidative stress and protein aggregation [18]. These findings highlight NA6 as a marine-derived oligosaccharide with therapeutic potential that may act in a context- and cell-type-dependent manner. However, its effects in crystal-induced inflammation and on *Ccl2* have not been investigated.

In this study, we employed an acute MSU-induced paw inflammation model in mice—a well-established surrogate of acute gout flares [19,20]—together with RAW264.7 macrophages in vitro to investigate whether topical NA6 could alleviate MSU-induced inflammation. Through integrated transcriptomic and network analyses, we identified *Ccl2* as the chemokine most strongly associated with the anti-inflammatory effect of NA6. Our findings provide new mechanistic insights into the anti-inflammatory effects of this red algae-derived oligosaccharide and support its further investigation for the topical management of gout flares.

## 2. Materials and Methods

### 2.1. Materials

Neoagarohexaose (NA6, purity 92.34%) was prepared and characterized in our laboratory according to our previously published protocol [21]. The structural identity and purity of NA6 were verified by high-performance liquid chromatography (HPLC) and electrospray ionization mass spectrometry (ESI-MS), as detailed in our previous study [22]. The NA6 used in the present study was obtained from the same batch as that characterized in our previous study [22]; the batch-specific HPLC chromatogram and ESI-MS spectrum of this batch are openly accessible in that publication [22], where the identity and purity of the exact material used here can be verified. The remaining 7.66% of the preparation consisted of minor fractions of other neoagaro-oligosaccharides of different degrees of polymerization that co-purify with NA6. The endotoxin content of the NA6 preparation was below 0.05 EU/mg, as determined by a chromogenic Limulus amebocyte lysate (LAL) assay, and endotoxin-free water, reagents, and consumables were used throughout. NA6 stock solutions were prepared in endotoxin-free PBS, filter-sterilized (0.22 μm), aliquoted, and stored at −80 °C until use.

Diclofenac diethylamine emulgel (Voltaren^®^, 1% *w*/*v*) was purchased from GlaxoSmithKline (Zürich, Switzerland). Dulbecco’s modified Eagle’s medium (DMEM), fetal bovine serum (FBS), and antibiotics (penicillin and streptomycin) were obtained from Gibco (Grand Island, NY, USA). Recombinant mouse CCL2 protein was purchased from PeproTech (Rocky Hill, NJ, USA). Antibodies against IL-6, IL-1β, and TNF-α for immunofluorescence were obtained from Aifang Biotechnology (Wuhan, China) and used at a 1:200 dilution. ELISA kits for TNF-α, IL-6, IL-1β, and CCL2 were purchased from Proteintech (Rosemont, IL, USA) (catalogue numbers KE10002, KE10007, KE10003, and KE10006 for TNF-α, IL-6, IL-1β, and CCL2, respectively).

### 2.2. Animals

C57BL/6J mice (male, 6–8 weeks old, body weight 22–26 g) were purchased from Fuzhou Wu’s Laboratory Animal Trade Co., Ltd. (Wuhan, China). Animals were housed in the Laboratory Animal Center of the Technology Innovation Center for Exploitation of Marine Biological Resources under a 12 h light/dark cycle at 22 ± 2 °C with 50–60% relative humidity, with food and water available ad libitum; five mice were housed per cage, and all mice were acclimatized for one week before the experiments. Only male mice were used to avoid the influence of estrous-cycle-related hormonal fluctuations on inflammatory responses and to maintain consistency with previous studies using this model [19]. A total of 30 mice were used in this study. The group size (*n* = 6 per group) was chosen based on previous studies using the same MSU model [19,20] and our preliminary experiments, balancing statistical power against the 3Rs principle; no formal a priori sample-size calculation was performed, which is acknowledged as a limitation. Mice were randomly allocated to the experimental groups using a computer-generated random sequence, and allocation was performed by an investigator not involved in outcome assessment. Paw thickness measurement, histological field selection and counting, fluorescence quantification, and data analysis were performed by investigators blinded to group allocation. The experimental unit was the individual animal. Mice in which MSU injection failed (no visible paw swelling at 2 h) were excluded before treatment allocation; no animals were excluded after allocation. At the endpoint, blood was collected from the retro-orbital sinus under deep isoflurane anesthesia, and mice were then euthanized by cervical dislocation. Humane endpoints were predefined (≥20% body-weight loss, severe ulceration, or persistent inability to reach food or water); no animal reached a humane endpoint. All experiments were conducted with the approval of the Laboratory Animal Management and Welfare Ethical Review Committee of the Third Institute of Oceanography (No. TIO-IACUC-03-2025-07-09).

### 2.3. MSU-Induced Paw Inflammation Model Establishment and Drug Administration

MSU crystals were suspended in endotoxin-free PBS at a concentration of 42.86 mg/mL, and the suspension was vortexed immediately prior to injection to ensure uniform dispersion. Under 2.5% isoflurane inhalation anesthesia, 70 µL of the MSU suspension (3 mg) was injected subcutaneously into the dorsal face of the right hind paw using a 26-gauge needle. Successful model establishment was confirmed by the onset of paw swelling within 2 h post-injection, which progressively increased and reached peak severity at approximately 24 h, followed by gradual resolution over 7 days [19,20].

To evaluate the anti-inflammatory efficacy of NA6 while maintaining route consistency, mice were randomly allocated to the following five experimental groups (*n* = 6 per group): (1) Sham group: received subcutaneous PBS injection in the right hind paw and topical PBS application (100 µL per paw) at 0, 6, 12, and 18 h; (2) MSU + Vehicle group: received MSU injection in the right hind paw and topical PBS application (100 µL per paw) at 0, 6, 12, and 18 h, serving as the model control; (3) MSU + NA6 5 mg/kg group: received MSU injection in the right hind paw and topical application of NA6 solution at 5 mg/kg (100 µL per paw) at 0, 6, 12, and 18 h; (4) MSU + NA6 10 mg/kg group: received MSU injection in the right hind paw and topical application of NA6 solution at 10 mg/kg (100 µL per paw) at 0, 6, 12, and 18 h; and (5) MSU + Diclofenac group: received MSU injection in the right hind paw and topical application of 1% (*w*/*v*) diclofenac diethylamine emulgel (Voltaren^®^, 100 µL per paw) at 0, 6, 12, and 18 h. All treatments were administered exclusively to the right (MSU-injected) paw. NA6 was dissolved in endotoxin-free PBS, and the applied concentration was adjusted for each mouse according to its body weight so that the intended dose was delivered in a fixed volume of 100 µL (for a representative 25 g mouse, the applied concentrations were 1.25 and 2.5 mg/mL for the 5 and 10 mg/kg groups, respectively, corresponding to approximately 0.125 and 0.25 mg per paw per application). The stated doses refer to each application; with four applications over 24 h (0, 6, 12, and 18 h), the cumulative 24-h doses were 20 and 40 mg/kg, respectively. Because the first NA6 application was performed at 0 h, immediately after MSU injection and before overt inflammation was established, the present regimen represents a prophylactic/very-early-intervention setting rather than treatment of an established flare. It should also be noted that the positive control was a commercial emulgel whereas the NA6 and vehicle groups received aqueous PBS; the formulation was therefore not matched with respect to viscosity, occlusion, and residence time, which is acknowledged as a limitation. Following each topical application, mice were gently held for approximately 1 min to allow initial spreading and absorption and to reduce removal of the solution by grooming.

### 2.4. Determination of the Paw Swelling

Before and after MSU injection, a digital caliper with an accuracy of 0.1 mm was used to measure paw thickness. The degree of swelling was expressed as the percentage increase in paw thickness, calculated by the following formula: Percentage increase = (Thickness after injection − Baseline thickness)/Baseline thickness × 100%. Each mouse was measured three times, and the average value was calculated. The area under the curve (AUC) of the time-dependent swelling index over 0–24 h was calculated for each mouse using the trapezoidal rule; the AUC values were normalized to the mean AUC of the MSU + Vehicle group, which was set as 100%.

### 2.5. Serum Biochemical Assays

Blood samples were collected from mice at 24 h post-MSU injection (6 h after the final treatment) and allowed to clot for 30 min at room temperature, then centrifuged at 3500 rpm for 15 min to obtain serum. Serum myeloperoxidase (MPO) activity was measured using commercially available kits (Solarbio, Beijing, China) according to the manufacturer’s instructions. Serum levels of TNF-α, IL-1β, and IL-6 were measured using ELISA kits (Proteintech, Rosemont, IL, USA) following the manufacturer’s protocols. Absorbance was measured at the indicated wavelengths using a microplate reader (Thermo Scientific, Waltham, MA, USA).

### 2.6. Histology and Immunofluorescence

Mouse paw tissues from the injected region were harvested, fixed in 4% paraformaldehyde, and embedded in paraffin. Sections (4 μm) were stained with hematoxylin and eosin (H&E) to evaluate subcutaneous tissue morphology and inflammatory cell infiltration. For immunofluorescence analysis, sections were deparaffinized, rehydrated, and subjected to antigen retrieval. After blocking with 5% BSA, sections were incubated with primary antibodies against IL-6, IL-1β, or TNF-α overnight at 4 °C, followed by incubation with fluorescently labeled secondary antibodies. Nuclei were counterstained with DAPI. Images were acquired using a fluorescence microscope (Zeiss, Oberkochen, Germany) with identical exposure and gain settings for all sections, and fluorescence intensities were quantified using ImageJ 1.8.0 software after background subtraction with identical threshold settings for all images. For each animal, three non-consecutive sections were examined, and five fields per section were randomly selected at a fixed anatomical depth in the subcutaneous tissue beneath the injection site by an investigator blinded to group allocation; field-level values were averaged to yield a single value per animal, which served as the statistical unit. The number of infiltrated inflammatory cells in the subcutaneous tissue was counted in five randomly selected high-power fields per section.

### 2.7. RNA Sequencing (RNA-Seq) and Bioinformatics Analysis

At 24 h post-MSU injection, mice were euthanized, and paw tissues from the injected region were promptly harvested using a sterile scalpel. For each mouse, the injected paw was divided: one portion was fixed in 4% paraformaldehyde for histology and immunofluorescence, and the remaining tissue was used for RNA extraction (RNA-seq and qRT-PCR). The three mice per group used for RNA-seq were randomly selected from the six mice of the respective group. The tissues were rinsed with RNase-free water, and non-target tissues (connective and adipose tissues) were excised. The tissues were then cut into small pieces, snap-frozen in liquid nitrogen, and stored at −80 °C until RNA extraction. RNA isolation, library preparation, and sequencing were performed by Metware (Wuhan, China). Total RNA was extracted according to the manufacturer’s protocol and quantified using a Qubit 4.0 fluorometer (Thermo Fisher Scientific, Waltham, MA, USA), and RNA integrity was assessed with a Qsep400 analyzer (BiOptic, New Taipei City, Taiwan). Poly(A)+ mRNA was enriched with oligo(dT) beads, fragmented, and reverse transcribed to cDNA. After end repair, A-tailing, adapter ligation, and PCR amplification, libraries were quality-checked and sequenced on an Illumina platform.

For bioinformatic analysis, raw reads were filtered with fastp to obtain clean data, which were aligned to the mouse reference genome (GRCm39, Ensembl annotation) using HISAT2, and gene-level read counts were generated with featureCounts (Subread); per-sample sequencing quality metrics (clean reads, Q30, and mapping rates) are provided in Supplementary Table S1; software versions were fastp v0.23.4, HISAT2 v2.2.1, featureCounts (Subread v2.0.6), DESeq2 v1.42.0, and clusterProfiler v4.10.1 (R v4.3.3). Gene expression levels were quantified, and differentially expressed genes (DEGs) were identified with DESeq2 using a negative binomial generalized linear model, with criteria of |log_2_FC| > 1 and Benjamini–Hochberg-adjusted *p* (FDR) < 0.05. Principal component analysis (PCA) was performed on variance-stabilized counts to assess sample-level clustering and quality. Gene Set Enrichment Analysis (GSEA) was performed using clusterProfiler against the Hallmark gene sets of the Molecular Signatures Database (MSigDB, mouse orthologues), with *p* values adjusted by the Benjamini–Hochberg method. Protein-protein interaction (PPI) network analysis was conducted using the STRING database (Mus musculus; minimum combined confidence score 0.4) and visualized with Cytoscape (version 3.10.2). The integrated target prioritization score was computed for each hub gene as the sum of three min–max-normalized components—statistical significance (−log_10_ adjusted *p*), effect size (|log_2_FC|), and node degree in the PPI network—with equal weighting (1/3 each); sensitivity to the weighting scheme was not formally assessed and is acknowledged as a limitation. Hub genes were identified based on the combined scores integrating expression significance, fold change, and network topology.

### 2.8. Cell Culture

RAW264.7 macrophages (Procell Life Science & Technology Co., Ltd., Wuhan, China) were cultured in DMEM supplemented with 10% FBS, 100 U/mL penicillin, and 0.1 mg/mL streptomycin. Cells were maintained at 37 °C in a humidified atmosphere of 5% CO_2_ and 95% air. Cells between passages 5 and 20 were used for all experiments; the cell line was routinely tested negative for mycoplasma contamination by PCR-based detection.

Cell viability assay. RAW264.7 macrophages were seeded in 96-well plates at a density of 1 × 10⁴ cells per well and allowed to attach overnight. Cells were then treated with NA6 (0.1, 0.25, 0.5, 1.0, and 1.5 mg/mL) for 24 or 36 h. Cell viability was determined using the CCK-8 assay (Beyotime Biotechnology, Shanghai, China) according to the manufacturer’s instructions. Absorbance was measured at 450 nm using a microplate reader (Thermo Scientific, Waltham, MA, USA). Because the CCK-8 assay measures cellular metabolic (dehydrogenase) activity, the absorbance was used as a surrogate readout of viable cell mass and was not interpreted as a direct measure of cell number. In addition, cells were treated with MSU (500 μg/mL) alone for 18 h to determine whether MSU stimulation itself affects the CCK-8 signal.

### 2.9. MSU Stimulation and NA6 Treatment In Vitro

RAW264.7 macrophages were seeded in 6-well plates at a density of 3 × 10⁵ cells per well and allowed to attach overnight. Cells were pretreated with NA6 at indicated concentrations (0.1, 0.2, or 0.5 mg/mL) for 30 min, followed by stimulation with 500 μg/mL MSU (InvivoGen, France) for 18 h. Cells and culture supernatants were collected for subsequent RNA extraction, protein analysis, and ELISA. The in vitro NA6 concentration range (0.1–0.5 mg/mL) was selected on the basis of the viability results (Section 3.6). In the in vivo experiments, NA6 was applied topically as solutions of approximately 1.25–2.5 mg/mL (Section 2.3); the local tissue exposure at the application site is therefore expected to lie in the sub-mg/mL to low-mg/mL range, so the in vitro concentrations fall within a pharmacologically relevant range, although the actual tissue concentrations were not measured.

### 2.10. Quantitative Real-Time PCR Analysis (qRT-PCR)

Total RNA was extracted from RAW264.7 macrophages and mouse paw tissues using the FastPure Cell/Tissue Total RNA Isolation Kit V2 (Vazyme, Nanjing, China) according to the manufacturer’s instructions. First-strand cDNA was synthesized using 1 μg RNA and HiScript IV All-in-One Ultra RT SuperMix (Vazyme, Nanjing, China). qRT-PCR was performed on a LightCycler detection system (Roche, Indianapolis, IN, USA) using ChamQ Blue Universal SYBR qPCR Master Mix (Vazyme, Nanjing, China). Primer amplification efficiencies (90–110%) were verified using standard curves of serial cDNA dilutions, and single-peak melt curves were confirmed for all amplicons; no-template controls and no-reverse-transcription controls were included in each run and yielded no amplification. *Gapdh* was verified to be stably expressed across MSU and NA6 treatment conditions in both RAW264.7 cells and paw tissues before its use as the reference gene. Relative mRNA expression was calculated using the 2^−ΔΔCt^ method and normalized to *Gapdh*. The primer sequences are listed in Table 1.

### 2.11. Enzyme-Linked Immunosorbent Assay (ELISA)

RAW264.7 macrophages were seeded in 6-well plates and treated as described in Section 2.9. Culture supernatants were collected and centrifuged at 3000 rpm for 10 min at 4 °C to remove debris. The levels of TNF-α, IL-6, IL-1β, and CCL2 in the supernatants were measured using corresponding mouse ELISA kits (Proteintech, Rosemont, IL, USA) according to the manufacturers’ instructions; the detection ranges of the kits were 7.8–500 pg/mL (TNF-α, IL-6, and IL-1β) and 3.13–200 pg/mL (CCL2). Absorbance was measured at 450 nm using a microplate reader (Thermo Scientific, Waltham, MA, USA), and cytokine concentrations were calculated from standard curves.

### 2.12. Transwell Chemotaxis Assay

RAW264.7 macrophages were pretreated with NA6 (0.5 mg/mL) for 30 min and then stimulated with MSU (500 μg/mL) for 18 h. Conditioned media were collected and centrifuged at 3000 rpm for 10 min at 4 °C to remove cells and residual MSU crystals, and the same volume of conditioned medium was placed in the lower chambers of 24-well Transwell plates (8-μm pore size, Corning, New York, NY, USA) for all groups. Untreated RAW264.7 macrophages (1 × 10^5^ cells in 100 μL serum-free DMEM) were seeded into the upper chambers. After incubation for 4 h at 37 °C, non-migrated cells on the upper surface were removed with a cotton swab. Migrated cells on the lower surface were fixed with 4% paraformaldehyde and stained with 0.1% crystal violet. Images were captured under a light microscope, and migrated cells were counted in five randomly selected fields per well by an investigator blinded to group identity. For the rescue assay, rCCL2 (50 ng/mL; PeproTech, Rocky Hill, NJ, USA) was added to the NA6-treated conditioned media prior to the migration assay. We note that in this design the responder cells are exposed to the complete conditioned secretome together with any residual NA6; the assay therefore measures the net chemotactic activity of the conditioned medium rather than the isolated contribution of CCL2, which is acknowledged as a limitation.

### 2.13. Statistical Analysis

All in vitro experiments were performed with *n* = 6 independent cell samples per group; the animal (in vivo experiments) or the individual cell sample (in vitro experiments) served as the statistical unit, and technical subsamples (multiple fields per section) were averaged within each biological replicate before statistical analysis. Data are expressed as mean ± standard deviation (SD). Statistical analysis was performed using Prism 10.1.2 (GraphPad Software, San Diego, CA, USA). Paw swelling time-course data (2, 8, and 24 h) were analyzed by two-way repeated-measures ANOVA with treatment and time as factors, followed by Tukey’s post hoc test. Single-time-point comparisons among multiple groups were performed using one-way ANOVA followed by Tukey’s post hoc test, and comparisons between two groups were conducted using unpaired Student’s *t*-test. Normality and homogeneity of variance were assessed using the Shapiro–Wilk and Brown–Forsythe tests, respectively. *p* < 0.05 was considered statistically significant (* *p* < 0.05, ** *p* < 0.01, *** *p* < 0.001, **** *p* < 0.0001); ns indicates not significant.

## 3. Results

### 3.1. NA6 Alleviates Paw Swelling and Reduces Circulating Inflammatory Markers in MSU-Challenged Mice

**Figure 1:**
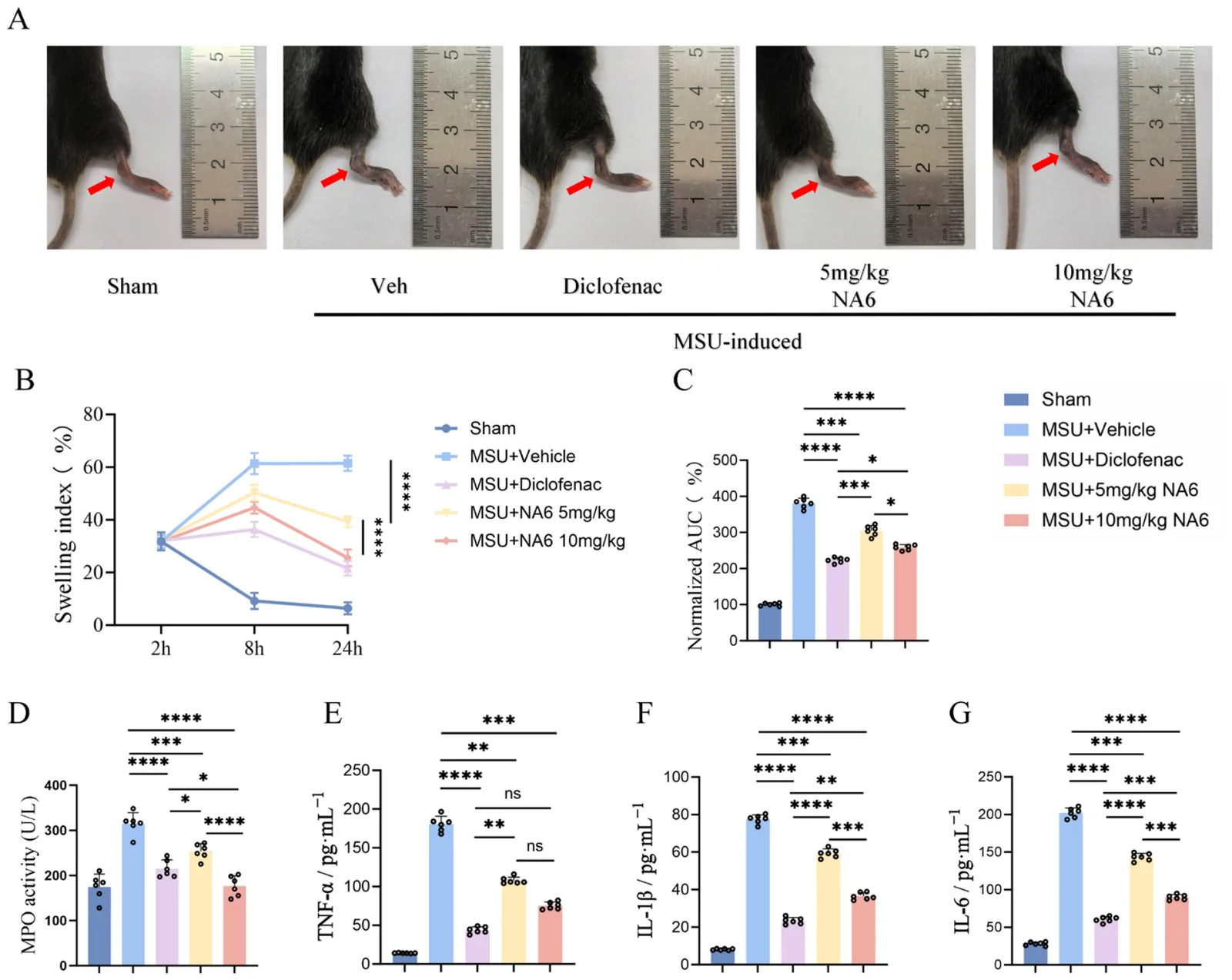
NA6 attenuates MSU-induced paw swelling and reduces circulating inflammatory markers. (**A**) Representative images of hind paws at 24 h post-MSU injection; the red arrow indicates the swollen region. (**B**) Paw swelling index monitored at 2, 8, and 24 h (two-way repeated-measures ANOVA with treatment and time as factors). (**C**) Quantification of swelling as the AUC of the swelling index over 0–24 h (trapezoidal rule), normalized to the MSU + Vehicle group mean (=100%). (**D**–**G**) Serum levels of MPO (**D**), TNF-α (**E**), IL-1β (**F**), and IL-6 (**G**). Data are mean ± SD (*n* = 6 mice); each symbol represents one mouse; brackets indicate the comparisons between the connected groups. * *p* < 0.05, ** *p* < 0.01, *** *p* < 0.001, **** *p* < 0.0001; ns, not significant.

Representative photographs of the hind paws (Figure 1A) showed the improvement in local edema after NA6 treatment. Compared with the MSU + Vehicle group, the paw swelling index (Figure 1B) and the corresponding area under the curve (AUC; Figure 1C) were significantly reduced in mice treated with NA6 (5 or 10 mg/kg). Under the present conditions, swelling in the NA6 10 mg/kg group did not differ significantly from that in the diclofenac emulgel group; we note, however, that this study was not designed or powered as an equivalence or non-inferiority trial. Additionally, the elevated serum MPO activity in the model group was significantly suppressed by NA6 administration (Figure 1D). Meanwhile, the increased serum levels of TNF-α (Figure 1E), IL-1β (Figure 1F), and IL-6 (Figure 1G) in MSU-challenged mice at 24 h were significantly reduced by NA6 treatment. These data indicate that topical NA6 alleviates local inflammatory edema and reduces circulating inflammatory markers at the 24-h peak of inflammation.

### 3.2. NA6 Attenuates MSU-Induced Paw Tissue Damage

**Figure 2:**
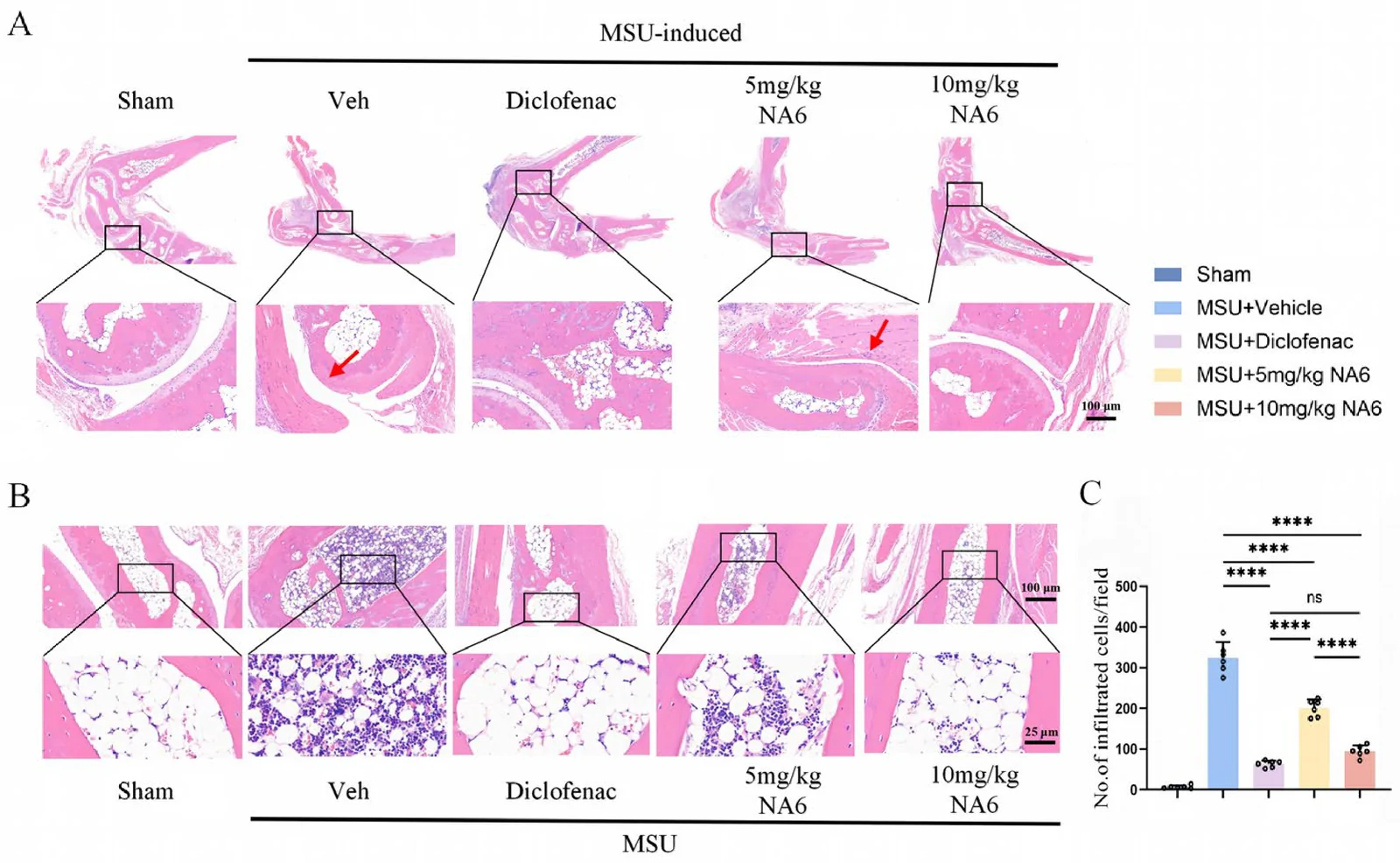
NA6 attenuates MSU-induced subcutaneous tissue damage. (**A**) H&E-stained paw sections; red arrows indicate structural disruption. (**B**) High-magnification images of inflammatory infiltration. (**C**) Quantification of infiltrated inflammatory cells per field. Scale bars apply to all panels of the same magnification; fields for counting were randomly selected at a fixed anatomical depth beneath the injection site by a blinded investigator, and field counts were averaged per mouse. Data are mean ± SD (*n* = 6 mice). **** *p* < 0.0001; ns, not significant.

Subcutaneous tissue damage was assessed via H&E staining of paw tissues. The MSU group exhibited prominent pathological alterations, including disrupted dermal and subcutaneous architecture, tissue edema, and disorganized cellular arrangement (Figure 2A). Extensive infiltration of inflammatory cells, including cells with granulocytic and mononuclear morphologies, was observed in the subcutaneous tissue of the MSU group (Figure 2B,C), indicating severe inflammatory injury; because H&E morphology alone does not reliably discriminate leukocyte lineages, we refrain from definitive cell-type assignment. In contrast, NA6-treated mice exhibited markedly improved tissue morphology, characterized by restored tissue integrity and substantially reduced numbers of infiltrating inflammatory cells, with cell counts not significantly different from those of the diclofenac-treated group.

### 3.3. NA6 Suppresses the Expression of Pro-Inflammatory Cytokines in Paw Tissue

**Figure 3:**
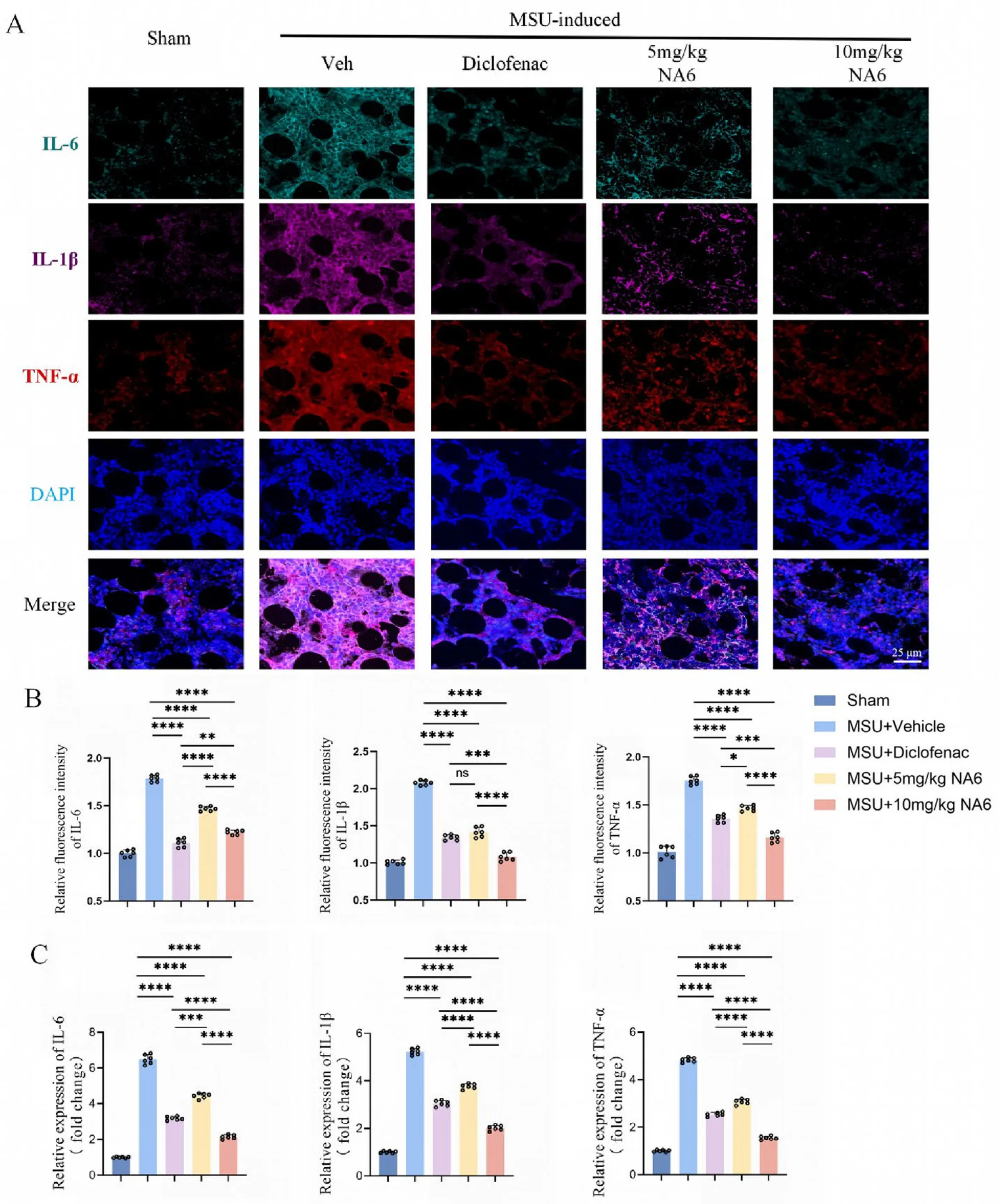
NA6 suppresses pro-inflammatory cytokine expression in paw tissue. (**A**) Immunofluorescence images of IL-6 (cyan), IL-1β (magenta), TNF-α (red), and DAPI (blue) in paw sections. (**B**) Quantification of relative fluorescence intensities. (**C**) Relative mRNA levels of *Il6*, *Il1b*, and *Tnf* determined by qPCR. Scale bars apply to all panels; acquisition settings were identical across groups; five randomly selected fields per section were quantified and averaged per mouse, with analysis performed blinded to group allocation. Data are mean ± SD (*n* = 6 mice). * *p* < 0.05, ** *p* < 0.01, *** *p* < 0.001, **** *p* < 0.0001; ns, not significant.

Immunofluorescence staining of paw tissues was performed to assess the local protein expression of inflammatory cytokines (Figure 3A). In the MSU group, strong positive fluorescence signals for IL-6, IL-1β, and TNF-α were detected. NA6 treatment markedly reduced these signals, with the high dose (10 mg/kg) producing the strongest reduction (Figure 3B). We note that, because fluorescence was quantified over whole tissue fields, the reduced signal may partly reflect the reduced inflammatory cellularity of the tissue in addition to reduced cytokine expression per cell. Consistent with these protein-level observations, qPCR analysis revealed that the mRNA levels of *Il6*, *Il1b*, and *Tnf* were significantly upregulated in the model group, whereas NA6 treatment markedly suppressed their mRNA expression, with the high dose again showing the strongest effect (Figure 3C). These results indicate that NA6 reduces local pro-inflammatory cytokine production in the affected paw tissues.

### 3.4. NA6 Attenuates MSU-Induced Inflammatory Transcriptional Changes in Paw Tissue

**Figure 4:**
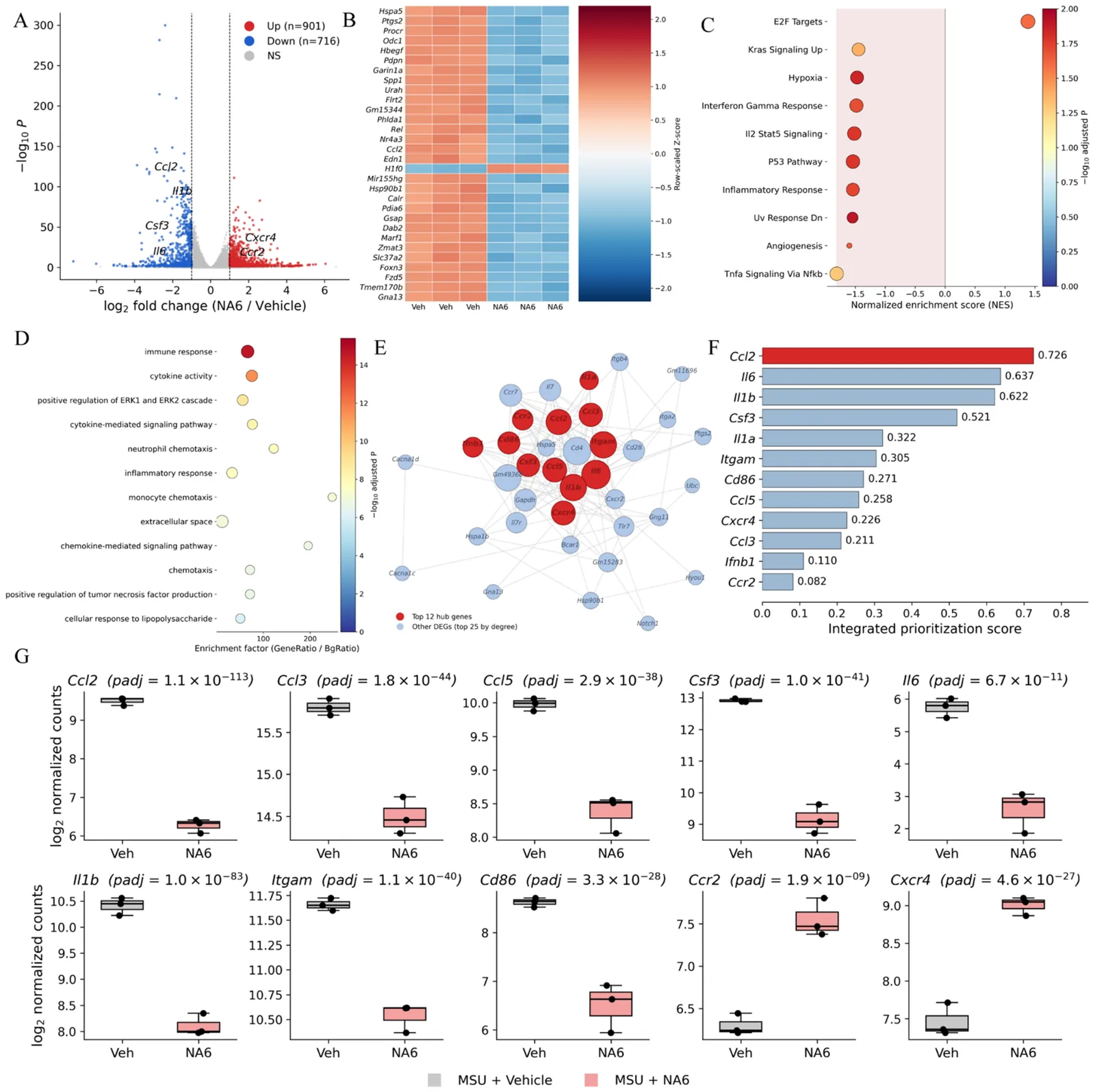
Transcriptomic and network analyses prioritize Ccl2 as the top-ranked NA6-responsive gene. RNA sequencing was performed on paw tissues from the MSU + Vehicle and MSU + NA6 (10 mg/kg) groups (*n* = 3 per group). (**A**) Volcano plot of DEGs (|log_2_FC| > 1, FDR < 0.05); red, up-regulated upon NA6 treatment; blue, down-regulated upon NA6 treatment. (**B**) Heatmap of the top 30 DEGs from the same contrast (row-scaled Z-scores), shown for visualization of group differences only. (**C**) GSEA dot plot of Hallmark gene sets; negative NES indicates enrichment in the MSU + Vehicle group (i.e., attenuation by NA6). (**D**) GO enrichment bubble plot of the 12 hub genes. (**E**) PPI network of the DEGs (STRING); red nodes indicate the top 12 hub genes. (**F**) Target prioritization scores integrating expression significance, fold change and network topology (min–max-normalized among the 12 hub genes). (**G**) Normalized expression of candidate genes from the same RNA-seq dataset (re-visualization; *n* = 3); DESeq2 adjusted *p* values are shown above each plot. PCA and sample-level quality-control plots are provided in Supplementary Figure S1.

RNA sequencing was performed on paw tissues from the MSU + Vehicle and MSU + NA6 (10 mg/kg) groups (*n* = 3 per group). Differential expression analysis (|log_2_FC| > 1, FDR < 0.05) revealed extensive transcriptomic differences upon NA6 treatment (Figure 4A), and the two groups showed distinct expression patterns of the top DEGs (Figure 4B). The genes most strongly suppressed by NA6 included the pro-inflammatory mediators *Ccl2*, *Ptgs2*, *Spp1*, *Edn1* and *Nr4a3*. Gene set enrichment analysis (GSEA) against the Hallmark collection showed that NA6 treatment was accompanied by broad attenuation of inflammation- and stress-related gene sets, including TNF-α signaling via NF-κB, inflammatory response, interferon-γ response, hypoxia, KRAS signaling and IL-2/STAT5 signaling, with all significantly enriched sets showing negative NES values (Figure 4C). Because RNA-seq was performed on bulk paw tissue without a sham-operated control group, and because the cellular composition of the paw changes markedly over the course of inflammation, these differences likely reflect, at least in part, the reduced immune-cell infiltration in NA6-treated paws rather than per-cell transcriptional reprogramming; we therefore interpret them as treatment-associated transcriptional changes.

### 3.5. Network Analysis Prioritizes Ccl2 as the Top-Ranked NA6-Responsive Gene

To prioritize candidate effectors associated with NA6 treatment, a protein–protein interaction (PPI) network of the DEGs was constructed using the STRING database, identifying 12 hub genes—*Ccl2*, *Il6*, *Il1b*, *Il1a*, *Csf3*, *Ccl3*, *Ccl5*, *Itgam*, *Ccr2*, *Cxcr4*, *Cd86* and *Ifnb1*—that highlight the central role of the chemokine–cytokine axis (Figure 4E). Functional enrichment of the hub genes converged on chemotaxis-related terms, including cell chemotaxis, chemokine-mediated signaling pathway, response to chemokine and leukocyte cell–cell adhesion (Figure 4D). An integrated target prioritization score combining expression significance, fold change and network topology ranked *Ccl2* first, ahead of *Il6* and *Il1b* (Figure 4F). Normalized RNA-seq expression data from the same dataset showed that *Ccl2*, *Ccl3*, *Ccl5*, *Csf3*, *Il6*, *Il1b*, *Itgam* and the macrophage activation marker *Cd86* were significantly lower in NA6-treated paws, whereas *Ccr2*, the cognate receptor of CCL2, and *Cxcr4* were higher (Figure 4G). We emphasize that Figure 4G is a re-visualization of the RNA-seq data and does not constitute independent experimental validation; independent qRT-PCR validation of *Ccl2* in paw tissue from the full cohort is provided in Supplementary Figure S2. Collectively, these results prioritize *Ccl2* as the top-ranked candidate effector associated with the anti-inflammatory action of NA6.

### 3.6. NA6 Inhibits MSU-Induced Inflammatory Responses and Macrophage Chemotaxis In Vitro

**Figure 5:**
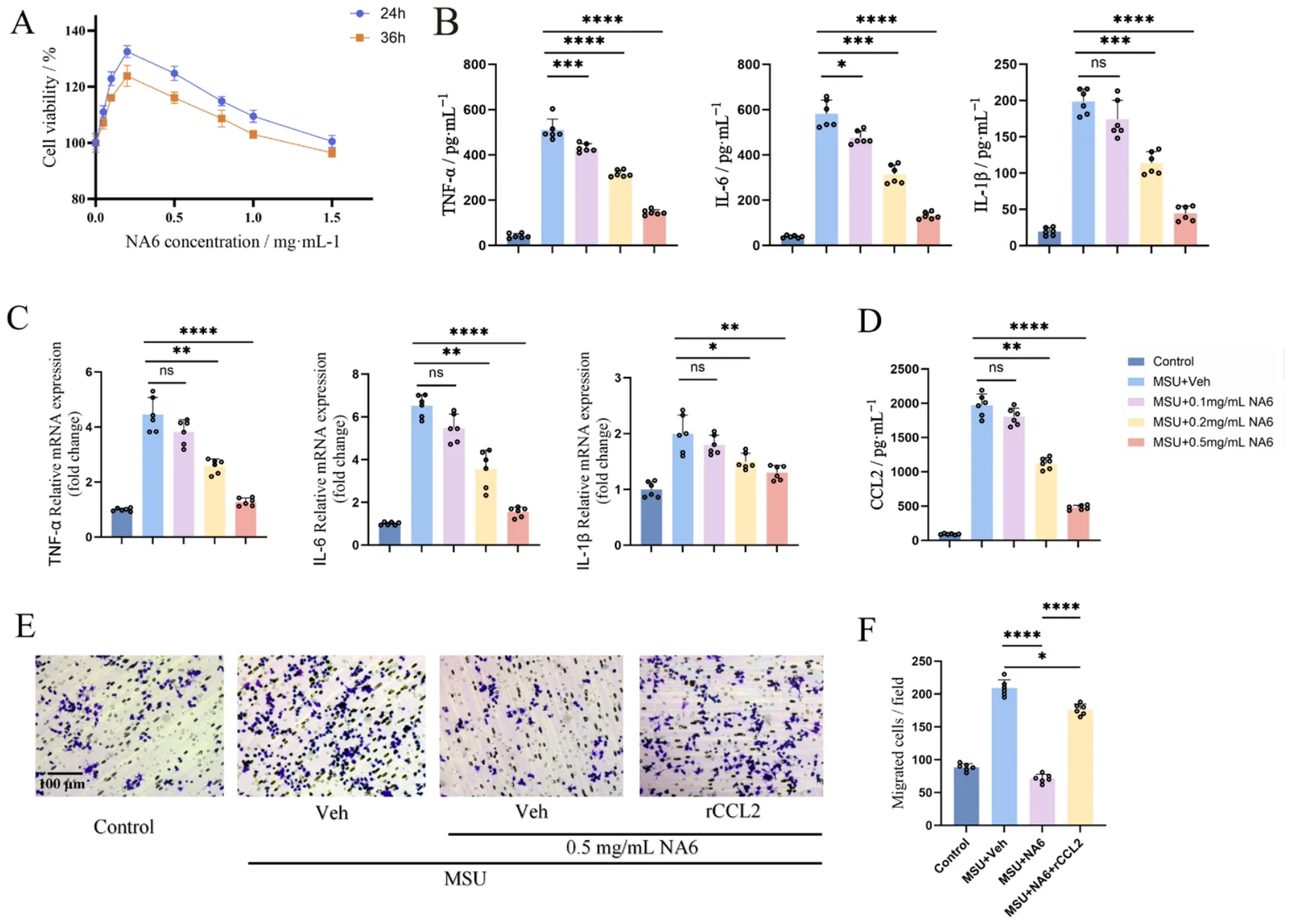
NA6 inhibits MSU-induced inflammation and macrophage chemotaxis in vitro. (**A**) CCK-8 metabolic activity. (**B**,**C**) Secretion and mRNA expression of TNF-α, IL-6, and IL-1β. (**D**) CCL2 secretion. (**E**,**F**) Transwell migration assay with rCCL2 rescue. For the in vitro panels (**A**–**F**), data are mean ± SD from *n* = 6 independent cell samples per group. Migrated-cell counting was performed blinded to group identity. * *p* < 0.05, ** *p* < 0.01, *** *p* < 0.001, **** *p* < 0.0001; ns, not significant.

RAW264.7 macrophages were treated with NA6 (0.1–1.5 mg/mL) for 24 or 36 h. As shown in Figure 5A, NA6 showed no detectable cytotoxicity at any tested concentration, with CCK-8 signals maintained above 90% of the control even at 1.5 mg/mL; a moderate increase in the CCK-8 signal was observed at low NA6 concentrations (0.1–0.5 mg/mL), which—given that the CCK-8 assay measures metabolic activity—was not interpreted as evidence of increased cell number. In addition, MSU alone (500 μg/mL, 18 h) did not significantly affect the CCK-8 signal (Supplementary Figure S3). The concentration range of 0.1–0.5 mg/mL was therefore selected for subsequent MSU stimulation experiments. NA6 suppressed MSU-induced protein secretion and mRNA expression of TNF-α, IL-6, and IL-1β in a concentration-related manner (Figure 5B,C), and NA6 treatment was accompanied by significantly reduced CCL2 secretion (Figure 5D). In a Transwell assay using conditioned media from 0.5 mg/mL NA6-treated cells, NA6 treatment was accompanied by reduced macrophage migration, and exogenous addition of recombinant CCL2 (rCCL2, 50 ng/mL) partially reversed this reduction (Figure 5E,F). We note that the rescue concentration exceeded the endogenous CCL2 levels measured in the conditioned media (approximately 0.5–2 ng/mL; Figure 5D) and that the conditioned medium contains the complete secretome together with residual NA6; these data therefore support an association between reduced CCL2 availability and reduced chemotaxis but do not establish CCL2 as the direct or sole mediator. Collectively, these in vitro findings are consistent with the in vivo and transcriptomic data, showing that the anti-inflammatory effect of NA6 is accompanied by reduced cytokine production and reduced CCL2-associated chemotactic activity.

## 4. Discussion

Gout flares are driven by a self-perpetuating cycle of MSU crystal-induced inflammation, with chemokine-mediated recruitment of inflammatory cells serving as a critical pathogenic mechanism [10,11]. In this study, we evaluated the anti-inflammatory effects of NA6, an oligosaccharide derived from marine red algae, in an acute MSU-induced paw inflammation model—a widely used surrogate of acute gout flares—and identified *Ccl2* as the chemokine most strongly associated with NA6 treatment. Topical administration of NA6 significantly alleviated paw swelling, reduced circulating MPO activity and pro-inflammatory cytokine levels at 24 h, and improved subcutaneous tissue histopathology. Transcriptomic profiling corroborated these findings, revealing broad attenuation of the MSU-induced inflammatory transcriptional signature. Specifically, GSEA indicated attenuation of TNF-α/NF-κB and IL-6-JAK-STAT3 signaling pathways, while PPI network analysis identified *Ccl2*, *Il1b*, and *Il6* as hub genes, with *Ccl2* ranked first upon integrated scoring. In vitro experiments further showed that NA6 reduced MSU-induced *Ccl2* expression and CCL2 secretion, which was accompanied by reduced chemotactic migration of macrophages. Collectively, these findings demonstrate that the anti-inflammatory effect of topical NA6 in this model is accompanied by reduced CCL2 production and CCL2-associated chemotactic activity.

CCL2 plays an essential role in the pathogenesis of gout by orchestrating the infiltration of monocytes and macrophages into inflamed tissues [11]. Upon MSU stimulation, resident tissue cells and infiltrating macrophages secrete large amounts of CCL2, which binds to CCR2 on circulating monocytes, directing their migration into the inflamed tissue and amplifying the local inflammatory cascade [11]; consistently, MSU-recruited monocytes have been shown to differentiate into CCL2-producing pro-inflammatory macrophages in murine models of gout [14]. The infiltrated macrophages subsequently release abundant pro-inflammatory cytokines including IL-1β, TNF-α, and IL-6, which further stimulate CCL2 production from surrounding cells, creating a positive feedback loop that sustains local inflammation [13]. In our study, NA6 treatment was accompanied by reduced CCL2 production at both the transcriptional and protein levels in MSU-stimulated macrophages. This reduction was accompanied by decreased expression of downstream inflammatory mediators including IL-1β, IL-6, and TNF-α in paw tissues, as well as reduced infiltration of inflammatory cells, indicating that NA6 treatment is associated with an interruption of the CCL2-driven amplification of local inflammation.

The association between NA6-mediated CCL2 reduction and chemotaxis was further supported by the chemotaxis assays. Conditioned media from MSU-stimulated macrophages potently promoted the migration of RAW264.7 macrophages, whereas NA6 treatment significantly attenuated this chemotactic activity. Exogenous supplementation with recombinant CCL2 partially reversed the reduction in cell migration, which is consistent with a contribution of CCL2 to the chemotactic activity of the conditioned media. We emphasize, however, that the rescue concentration used (50 ng/mL) exceeds the endogenous CCL2 concentrations measured in the conditioned media (approximately 0.5–2 ng/mL), and that pathway-specific interventions—such as CCL2 neutralization, CCR2 blockade, or genetic perturbation—were not performed; the present data therefore do not establish CCL2 as the direct or primary mediator of NA6 activity. This interpretation is consistent with evidence that suppressing CCL2 production or blocking CCL2/CCR2 signalling reduces monocyte recruitment and inflammatory tissue damage in gout and other arthritis models [23,24], and with the demonstrated role of CCL2 in MSU-driven monocyte recruitment [14].

The transcriptomic and network analyses provided an unbiased overview of the transcriptional changes accompanying NA6 treatment. While GSEA revealed broad attenuation of classical inflammatory pathways such as TNF-α/NF-κB and IL-6-JAK-STAT3, these changes likely reflect the overall resolution of the inflammatory environment rather than direct inhibition of these specific signaling cascades by NA6. We therefore focused on the chemokine network. The PPI network and integrated scoring system identified *Ccl2* as the top-ranked NA6-responsive gene, while *Il1b* and *Csf3* occupied closely related regulatory nodes. From a pathophysiological perspective, the coordinated reduction of these three hub genes is noteworthy: *Ccl2* is essential for recruiting monocytes and macrophages to the inflamed tissue, whereas CSF3 drives neutrophil production and mobilization, a process central to granulopoiesis and acute inflammatory responses [25]. Furthermore, IL-1β—a master pro-inflammatory cytokine in gout—acts as a potent stimulator of CCL2 production, establishing a positive feedback loop that amplifies local inflammation [13,26]. The concurrent attenuation of *Ccl2*, its upstream inducer *Il1b*, and the neutrophil mobilizer *Csf3* is consistent with the observed reduction of inflammatory cell infiltration and provides a plausible mechanistic framework for the anti-inflammatory effect of NA6 in this model.

The present study extends our recent work showing joint-protective effects of topically applied NA6 in MIA/CIOA mouse models of osteoarthritis, which acted primarily through antioxidant pathways and cartilage protection [22]. We emphasize that the two studies are independent: they used different disease models (acute subcutaneous MSU challenge versus chronic intra-articular MIA/CIOA osteoarthritis), different animal cohorts, different endpoints, and entirely separate samples, datasets, and images; no animals, raw data, or images are shared between the two studies, although the NA6 material was obtained from the same characterized batch. The novelty of the present work lies specifically in the acute MSU-induced inflammation setting and in the observed association between NA6 treatment, reduced *Ccl2* expression, and reduced chemotactic activity—not in the general claim that topical NA6 is anti-inflammatory. The distinct mechanistic emphasis in the two settings—acute chemokine-driven inflammatory influx here versus chronic oxidative degeneration in osteoarthritis [22]—suggests that NA6 may engage context-dependent mechanisms in different types of joint-related inflammation.

Several marine-derived natural products have previously been investigated for their anti-inflammatory effects in gout models, providing an important background for evaluating NA6. For instance, alginate oligosaccharides have been shown to alleviate MSU-induced joint inflammation primarily through suppression of the NLRP3 inflammasome and reduction of ROS production [27]. Fucoidan also exhibits anti-inflammatory properties in arthritis models [28]; however, its high molecular weight often limits oral bioavailability and topical skin penetration. The moderate molecular weight (0.94 kDa) and high water solubility of NA6 make it suitable for local aqueous application, although its transdermal absorption and tissue penetration remain to be experimentally validated. Distinct from these reported marine compounds, our data point to an association of NA6 with the chemokine axis: rather than directly inhibiting inflammasome activation, NA6 treatment was accompanied by reduced CCL2 production and attenuated chemotactic recruitment of inflammatory cells into the subcutaneous tissue, which was reflected in the in vivo outcomes of reduced paw swelling, circulating MPO activity, and inflammatory cell infiltration.

In comparison to existing pharmacological interventions for gout flares, NA6 may offer potential advantages. Current first-line therapies including NSAIDs, colchicine, and glucocorticoids primarily target downstream inflammatory mediators or general immune suppression and are frequently accompanied by gastrointestinal, renal, or metabolic adverse effects [7]. In contrast, NA6 treatment is associated with modulation of chemokine-driven recruitment of inflammatory cells, an upstream event in the pathogenic cascade. The topical route employed in this study is intended to achieve high local drug concentrations at the application site; however, skin penetration, local retention, and systemic exposure of topically applied NA6 were not measured in this study, and the aqueous PBS vehicle was not formulation-matched to the commercial diclofenac emulgel; these translational aspects therefore remain to be established. The favorable safety profile of agarose-derived oligosaccharides reported previously [15] nevertheless supports further investigation of NA6 for topical application.

Interestingly, while NA6 shares structural features with other marine oligosaccharides known for their anti-inflammatory properties—such as alginate oligosaccharides [27] and fucoidan [28]—our findings reveal a distinct association with *Ccl2*. Yin et al. reported that alginate oligosaccharides ameliorate MSU-induced joint inflammation through suppression of NLRP3 inflammasome activation [27], whereas our transcriptomic data indicate that the most prominent change accompanying NA6 treatment is the attenuation of chemokine-mediated inflammatory cell trafficking. Whether NA6 also modulates the NLRP3–ASC–caspase-1 axis—the canonical pathway of MSU-induced inflammation [26]—was not examined in the present study and represents an important question for future work. It is also noteworthy that NA6 has previously been described as a TLR4 agonist capable of activating dendritic cells and enhancing antiviral and antitumor immunity [16,17]; the anti-inflammatory phenotype observed here is not necessarily contradictory, because TLR4-mediated responses are cell-type- and dose-dependent, the immune-enhancing effects were observed in dendritic-cell/NK-cell contexts at defined doses, and the present study examined MSU-activated macrophages under endotoxin-controlled conditions. This difference may also reflect the unique oligosaccharide composition and chain length of NA6, which could determine its specific interactions with cellular receptors or uptake mechanisms. Future studies comparing the structure-activity relationships of different marine oligosaccharides may provide further insights into their divergent mechanistic preferences.

Several limitations of this study should be acknowledged. First, we used an acute, subcutaneous dorsal-paw MSU inflammation model, which is a widely accepted surrogate of acute gout flares [19,20] but does not recapitulate intra-articular pathology; no synovial, cartilage, or joint endpoints were assessed, and validation in a genuine intra-articular model is required before conclusions on joint protection can be drawn. Second, because NA6 was first applied immediately after MSU injection, the regimen is prophylactic rather than therapeutic, and no nociceptive or functional endpoints were measured; delayed-treatment designs and pain-related outcomes should be evaluated in future studies. Third, although network analysis prioritized *Ccl2* as the top-ranked NA6-responsive gene, the evidence remains associative: the rCCL2 rescue concentration was supraphysiological, no CCL2 loss-of-function, neutralization, or CCR2-blockade experiments were performed, and the Transwell design exposes responder cells to the complete conditioned secretome and residual NA6; whether CCL2 is the direct or primary mediator of NA6 activity remains to be established. Fourth, RNA-seq was performed on bulk paw tissue without a sham control group and with a limited sample size, so the observed transcriptional differences may partly reflect changes in tissue cellularity; the key gene *Ccl2* was therefore additionally validated by qRT-PCR in paw tissue from the full cohort, although the small RNA-seq sample size remains a limitation. Fifth, the pharmacokinetic properties of topically applied NA6, including transdermal absorption, penetration depth, and systemic exposure, were not characterized, and the vehicle was not formulation-matched to the diclofenac comparator. Sixth, serum inflammatory markers were assessed at a single time point, and the experiments used only male mice. Finally, the in vitro assays were limited to the RAW264.7 macrophage cell line; validation in primary macrophages (e.g., bone marrow-derived macrophages) or human monocytic cells (e.g., THP-1) would strengthen the translational relevance of our findings. Addressing these aspects in future studies will help establish the applicability of NA6 as a topical agent for gout-related inflammation.

## 5. Conclusions

In conclusion, this study demonstrates that topical application of neoagarohexaose (NA6), a well-defined oligosaccharide from red algal agarose, alleviates acute MSU-induced paw inflammation in mice, a widely used surrogate of acute gout flares. The anti-inflammatory effect is accompanied by reduced local and circulating inflammatory mediators, reduced *Ccl2* expression and CCL2 secretion, and reduced CCL2-associated macrophage chemotactic activity. Although pathway-specific causal evidence remains to be established, these findings support NA6 as a marine-derived candidate worthy of further investigation for the topical management of gout-related inflammation.

## Figures and Tables

**Table 1:** Sequences of primers used for qRT-PCR.

Gene (Mouse)	Primer	Sequence
*Il6*	Forward	TAGTCCTTCCTACCCCAATTTCC
	Reverse	TTGGTCCTTAGCCACTCCTTC
*Il1b*	Forward	TGGGTACTGGAGAGTGGTCA
	Reverse	GGCTTGGGAGTGAAGAGGTC
*Tnf*	Forward	CCTGTAGCCCACGTCGTAG
	Reverse	GGGAGTAGACAAGGTACAACCC
*Ccl2*	Forward	TTAAAAACCTGGATCGGAACCAA
	Reverse	GCATTAGCTTCAGATTTACGGGT
*Gapdh*	Forward	CCCTTCATTGACCTCAACTACATG
	Reverse	TGGGATTTCCATTGATGACAAGC

## Data Availability

The raw RNA sequencing data generated in this study have been deposited in the NCBI BioProject database under accession number PRJNA1348749. Processed data and additional information are available from the corresponding author upon reasonable request.

## Supplementary Materials

The following supporting information can be downloaded at: https://www.mdpi.com/article/10.3390/cimb48101074/s1.

## Abbreviations

The following abbreviations are used in this manuscript:

NA6	Neoagarohexaose
GA	Gouty arthritis
MSU	Monosodium urate
*Ccl2*	C-C motif chemokine ligand 2
*Ccr2*	C-C chemokine receptor type 2
rCCL2	Recombinant CCL2
MPO	Myeloperoxidase
TNF-α	Tumor necrosis factor-alpha
IL-1β	Interleukin-1 beta
IL-6	Interleukin-6
DEGs	Differentially expressed genes
GSEA	Gene set enrichment analysis
PPI	Protein-Protein Interaction

## References

[1] Yang Q.B., Zhang M.Y., Yang L., Wang J., Mi Q.S., Zhou J.G. (2024). Deficiency of histone deacetylases 3 in macrophage alleviates monosodium urate crystals-induced gouty inflammation in mice. Arthritis Res. Ther..

[2] Galozzi P., Bindoli S., Doria A., Oliviero F., Sfriso P. (2021). Autoinflammatory Features in Gouty Arthritis. J. Clin. Med..

[3] Gu H., Yu H., Qin L., Yu H., Song Y., Chen G., Zhao D., Wang S., Xue W., Wang L. (2023). MSU crystal deposition contributes to inflammation and immune responses in gout remission. Cell Rep..

[4] Dalbeth N., Merriman T.R., Stamp L.K. (2016). Gout. Lancet.

[5] Shi C., Zhou Z., Chi X., Xiu S., Yi C., Jiang Z., Chen R., Zhang L., Liu Z. (2023). Recent advances in gout drugs. Eur. J. Med. Chem..

[6] Peng X., Li X., Xie B., Lai Y., Sosnik A., Boucetta H., Chen Z., He W. (2023). Gout therapeutics and drug delivery. J. Control. Release Off. J. Control. Release Soc..

[7] Zeng X., Liu Y., Fan Y., Wu D., Meng Y., Qin M. (2023). Agents for the Treatment of Gout: Current Advances and Future Perspectives. J. Med. Chem..

[8] Hong Y., Boiti A., Vallone D., Foulkes N.S. (2024). Reactive Oxygen Species Signaling and Oxidative Stress: Transcriptional Regulation and Evolution. Antioxidants.

[9] Patergnani S., Bouhamida E., Leo S., Pinton P., Rimessi A. (2021). Mitochondrial Oxidative Stress and “Mito-Inflammation”: Actors in the Diseases. Biomedicines.

[10] Zhang S., Li D., Fan M., Yuan J., Xie C., Yuan H., Xie H., Gao H. (2024). Mechanism of Reactive Oxygen Species-Guided Immune Responses in Gouty Arthritis and Potential Therapeutic Targets. Biomolecules.

[11] Zhu S., Liu M., Bennett S., Wang Z., Pfleger K.D.G., Xu J. (2021). The molecular structure and role of CCL2 (MCP-1) and C-C chemokine receptor CCR2 in skeletal biology and diseases. J. Cell. Physiol..

[12] Moadab F., Khorramdelazad H., Abbasifard M. (2021). Role of CCL2/CCR2 axis in the immunopathogenesis of rheumatoid arthritis: Latest evidence and therapeutic approaches. Life Sci..

[13] Scanu A., Oliviero F., Gruaz L., Sfriso P., Pozzuoli A., Frezzato F., Agostini C., Burger D., Punzi L. (2010). High-density lipoproteins downregulate CCL2 production in human fibroblast-like synoviocytes stimulated by urate crystals. Arthritis Res. Ther..

[14] Martin W.J., Shaw O., Liu X., Steiger S., Harper J.L. (2011). Monosodium urate monohydrate crystal-recruited noninflammatory monocytes differentiate into M1-like proinflammatory macrophages in a peritoneal murine model of gout. Arthritis Rheum..

[15] Chen X., Fu X., Huang L., Xu J., Gao X. (2021). Agar oligosaccharides: A review of preparation, structures, bioactivities and application. Carbohydr. Polym..

[16] Kim M., Lee J.E., Cho H., Jung H.G., Lee W., Seo H.Y., Lee S.H., Ahn D.G., Kim S.J., Yu J.W. (2020). Antiviral efficacy of orally delivered neoagarohexaose, a nonconventional TLR4 agonist, against norovirus infection in mice. Biomaterials.

[17] Lee M.H., Jang J.H., Yoon G.Y., Lee S.J., Lee M.G., Kang T.H., Han H.D., Kim H.S., Choi W.S., Park W.S. (2017). Neoagarohexaose-mediated activation of dendritic cells via Toll-like receptor 4 leads to stimulation of natural killer cells and enhancement of antitumor immunity. BMB Rep..

[18] Zhang Q., Li J., Sun Y., Song S., Li X., Chen G. (2023). Neoagarohexaose Protects against Amyloid β-Induced Oxidative Stress and Aggregation. Appl. Biochem. Microbiol..

[19] Mariotte A., De Cauwer A., Po C., Abou-Faycal C., Pichot A., Paul N., Aouadi I., Carapito R., Frisch B., Macquin C. (2020). A mouse model of MSU-induced acute inflammation in vivo suggests imiquimod-dependent targeting of Il-1β as relevant therapy for gout patients. Theranostics.

[20] Schiltz C., Lioté F., Prudhommeaux F., Meunier A., Champy R., Callebert J., Bardin T. (2002). Monosodium urate monohydrate crystal-induced inflammation in vivo: Quantitative histomorphometric analysis of cellular events. Arthritis Rheum..

[21] Chen X., Lin H., Jin M., Zeng R., Lin M. (2019). Characterization of a novel alkaline β-agarase and its hydrolysates of agar. Food Chem..

[22] Wu N., Du Y., Wu C., Chan Z., Zeng R. (2026). Neoagarohexaose Attenuates Inflammatory and Oxidative Joint Injury in MIA/CIOA Mouse Models of Osteoarthritis. Int. J. Mol. Sci..

[23] Liu L., Xue Y., Zhu Y., Xuan D., Yang X., Liang M., Wang J., Zhu X., Zhang J., Zou H. (2016). Interleukin 37 limits monosodium urate crystal-induced innate immune responses in human and murine models of gout. Arthritis Res. Ther..

[24] Raghu H., Lepus C.M., Wang Q., Wong H.H., Lingampalli N., Oliviero F., Punzi L., Giori N.J., Goodman S.B., Chu C.R. (2017). CCL2/CCR2, but not CCL5/CCR5, mediates monocyte recruitment, inflammation and cartilage destruction in osteoarthritis. Ann. Rheum. Dis..

[25] Mehta H.M., Corey S.J. (2021). G-CSF, the guardian of granulopoiesis. Semin. Immunol..

[26] Kim S.K. (2022). The Mechanism of the NLRP3 Inflammasome Activation and Pathogenic Implication in the Pathogenesis of Gout. J. Rheum. Dis..

[27] Yin C., Lyu Q., Dong Z., Liu B., Zhang K., Liu Z., Yu Q., Li P., Wei Z., Tai Y. (2024). Well-defined alginate oligosaccharides ameliorate joint pain and inflammation in a mouse model of gouty arthritis. Theranostics.

[28] Sanjeewa K.K.A., Herath K.H.I.N.M., Yang H.-W., Choi C.S., Jeon Y.-J. (2021). Anti-Inflammatory Mechanisms of Fucoidans to Treat Inflammatory Diseases: A Review. Mar. Drugs.

