# Supplementary material for: Topical Neoagarohexaose from Red Algae Alleviates Acute MSU-Induced Paw Inflammation Accompanied by Reduced Ccl2-Associated Macrophage Chemotaxis
Source: Curr Issues Mol Biol. 2026 Oct 17;48(10):1074. doi: 10.3390/cimb48101074 (PMC13703483; doi:10.3390/cimb48101074)
Supplement: Supplementary file 1 [file cimb-48-01074-s001.zip › cimb-4552920-supplementary.pdf]

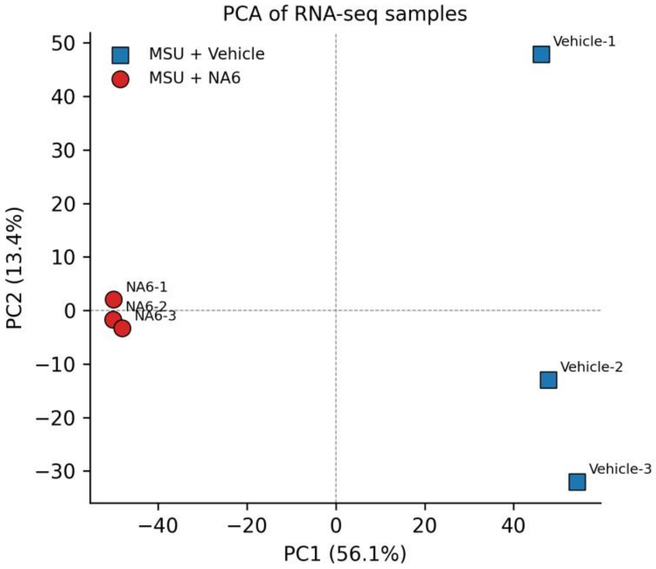

Figure S1: Principal component analysis (PCA) of paw-tissue RNA-seq samples. PCA was performed on variance-stabilized counts (DESeq2). Each symbol represents one mouse (n = 3 per group); the MSU + Vehicle and MSU + NA6 (10 mg/kg) groups separate along PC1 (56.1% of variance) and PC2 (13.4%), confirming distinct transcriptomic profiles and acceptable within-group clustering.

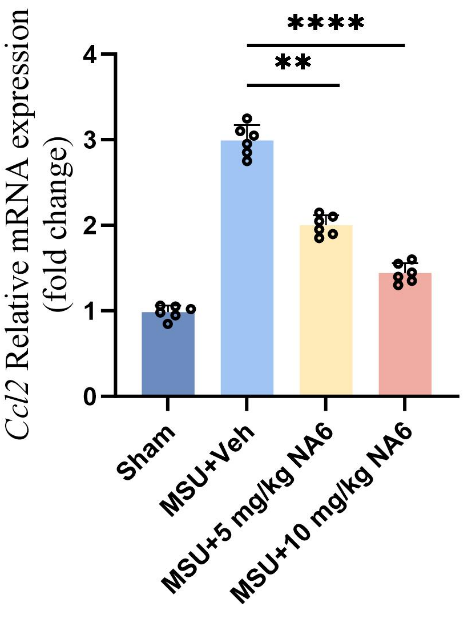

Figure S2: qRT-PCR validation of *Ccl2* mRNA expression in paw tissues from the full cohort (n = 6 mice per group). Total RNA was extracted from paw tissues of the four experimental groups and analyzed by qRT-PCR; *Ccl2* expression was normalized to *Gapdh* using the $2^{-\Delta\Delta Ct}$ method and expressed as fold change relative to the Sham group. Bars represent mean ± SD, and each symbol represents one mouse. $^{**}P < 0.01$, $^{****}P < 0.0001$ versus the MSU + Vehicle group (one-way ANOVA with Tukey's multiple comparisons test).

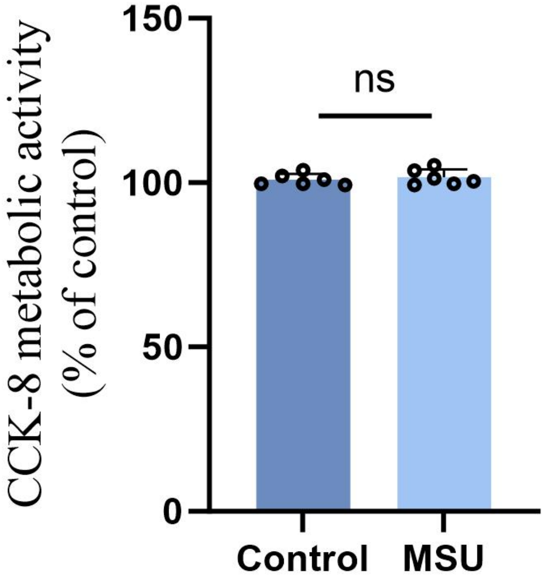

Figure S3: Effect of MSU alone on the CCK-8 signal in RAW264.7 macrophages. Cells were treated with MSU (500 μg/mL) or fresh medium (Control) for 18 h, and the CCK-8 absorbance (450 nm) was normalized to the Control group. Bars represent mean ± SD, and each symbol represents one independent cell sample (n = 6 per group). ns, not significant (unpaired two-tailed Student's t-test).

Table S1: Per-sample RNA-sequencing quality metrics. Raw reads were filtered with fastp to obtain clean reads, which were aligned to the mouse reference genome (GRCm39) using HISAT2. Q20/Q30: percentages of bases with Phred quality scores ≥ 20/30. Three biological replicates per group.

Sample	Raw reads	Clean reads	Clean bases (Gb)	Error rate (%)	Q20 (%)	Q30 (%)	GC content (%)	Total mapping rate (%)	Unique mapping rate (%)
MSU + NA6 (10 mg/kg)	68,927,290	66,785,496	10.02	0.01	98.37	95.01	48.33	95.26	88.96
MSU + NA6 (10 mg/kg)	68,876,976	67,068,234	10.06	0.01	98.28	94.75	48.65	94.89	88.55
MSU + NA6 (10 mg/kg)	73,609,688	71,308,176	10.7	0.01	98.47	95.29	49.51	94.97	88.74
MSU + Vehicle	58,105,788	55,963,910	8.39	0.01	98.33	94.93	47.86	94.45	88.12
MSU + Vehicle	68,261,754	65,690,632	9.85	0.01	98.39	95.09	48.08	94.34	87.85
MSU + Vehicle	60,942,392	58,825,580	8.82	0.01	98.41	95.17	47.93	94.32	88
